# ﻿Biodiversity of Nearctic inland water: discovery of the genus *Heterorotula* (Porifera, Spongillida, Spongillidae) in the Appalachian Mountains, with biogeographical implications and description of new species

**DOI:** 10.3897/zookeys.1110.79615

**Published:** 2022-07-04

**Authors:** Renata Manconi, John Copeland, Stan Kunigelis, Roberto Pronzato

**Affiliations:** 1 Dipartimento di Medicina Veterinaria, Università di Sassari, Via Vienna 2, 07100 Sassari, Italy; 2 Department of Biology, Lincoln Memorial University, 6965 Cumberland Gap Parkway, Harrogate, Tennessee, 37752, USA; 3 DeBusk College of Osteopathic Medicine, Lincoln Memorial University, 6965 Cumberland Gap Parkway, Harrogate, Tennessee, 37752, USA; 4 Dipartimento di Scienze della Terra, dell’Ambiente e della Vita, Università di Genova, Corso Europa 26, 16132 Genova, Italy

**Keywords:** Aquatic biodiversity, biogeography, evolutionary history, freshwater sponges, morphotraits, Tennessee, United States

## Abstract

This paper reports the discovery of a small population of sponges in the Pigeon River of eastern Tennessee, USA, which were morphologically distinct from Spongillida of North America. A morphological comparative analysis resulted in the first Nearctic record of the genus *Heterorotula* with the description of a new species *Heterorotulalucasi***sp. nov.** diverging from all other known species by its unique combinations of diagnostic morphotraits of spicules and gemmules. The new record enlarges the geographic range of the genus which has been known until now only from Australia, New Zealand, New Caledonia, Japan (as an alien species), and from subequatorial Brazil (as subfossil remains). The discovery of a biogeographic enclave of *Heterorotula* in the southeastern United States contributes to the understanding of Porifera inland water biodiversity, biogeographic patterns, and adaptive morphotraits in the Nearctic and globally. Data confirm that the Appalachian region (Ordovician–Permian origin) of Tennessee and, in general, of North America have high levels of diversity and endemicity.

## ﻿Introduction

The aquatic biodiversity of the southeastern United States is recognized as the most biologically rich on the North American continent (Elkins et al. 2019). Assessments of aquatic biodiversity have found the southeastern United States to have levels of diversity and endemism rivalling those of tropical regions (Abell 2000; Collen et al. 2014).

The mountains and valleys of the Appalachian region of North America extend for 3,200 km from the Canadian province of Newfoundland to central Alabama of the United States. There was no one-time orogeny creating the Appalachian Mountains; instead, these mountains were formed by several major and minor orogenies. Erosion has reduced ancient Appalachian peaks of over 5,000 m to slightly over 2,000 m today (Pickering et al. 2003).

The central and southern Appalachians did not experience Pleistocene glaciation. Their alignment north to south provided organisms a migration corridor to the south thereby avoiding extinctions (Pickering et al. 2003). Due to topography, climate, and being spared Pleistocene glaciation, the southern Appalachian Mountains are one of the most biologically diverse regions in the temperate world. High aquatic species diversity has led to this region being recognized as a global center of aquatic biodiversity (McLarney 1999; Curtin et al. 2002).

Until recently, little was known concerning the sponge fauna of the Appalachian region of Tennessee. However, including the species described in this article, 13 species have been documented from this region (Table 1) (Copeland et al. 2015, 2019, 2020). During July 2013 in the Pigeon River, a small population of sponges was discovered which was morphologically distinct from all other known extant Spongillida of North America. We report here on this population as the first record of the genus *Heterorotula* Penney & Racek, 1968 from the Nearctic Region and describe it as a new species.

**Table 1.** Checklist of the freshwater sponges (Porifera: Spongillida) of the Appalachian region of Tennessee (southeastern Nearctic Region) with the new species record (bold) from Pigeon River.

Family	Species
Potamolepidae	*Cherokeesiaarmata* Copeland, Pronzato & Manconi, 2015
Spongillidae	*Corvospongillabecki* Poirrier, 1978
	*Ephydatiafluviatilis* (Linnaeus, 1759)
	*Ephydatiamuelleri* (Lieberkuhn, 1855)
	*Eunapiusfragilis* (Leidy, 1851)
	*Heteromeyenialatitenta* (Potts, 1881)
	*Heteromeyeniatubisperma* ((Potts, 1881)
	***Heterorotulalucasi* sp. nov.**
	*Racekielaryderi* (Potts, 1882)
	*Radiospongillacerebellata* (Bowerbank, 1863)
	*Radiospongillacrateriformis* (Potts, 1882)
	*Radiospongillacrateriformis* (Potts, 1882)
	*Trochospongillahorrida* (Weltner, 1893)

## ﻿Study area

**Figure 1.**
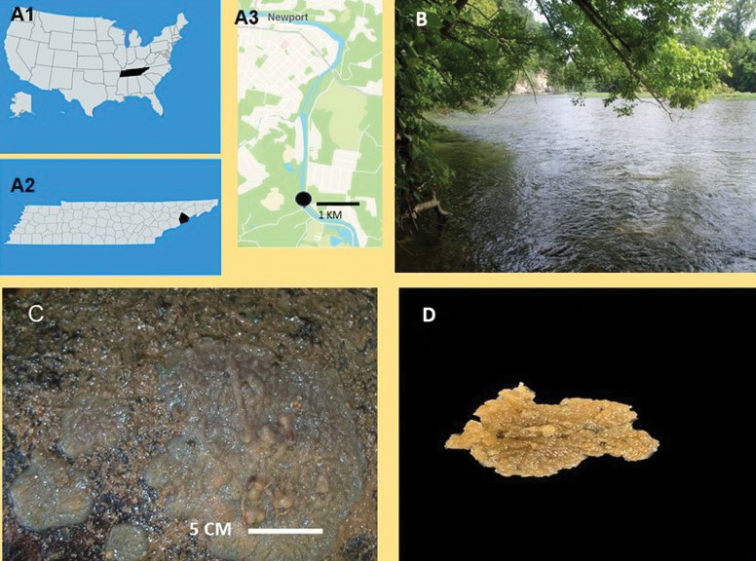
Study area **A1** Tennessee in the southeastern region of the United States **A2** location of Cocke County in Tennessee **A3** type locality of *Heterorotulalucasi* sp. nov. is indicated by a black circle in the Pigeon River (35.9396, −83.1786) **B** riparian habitat of the new sponge species at the type locality **C** macrophotograph *in vivo* of holotype (USNM 1662182) with tawny color and encrusting growth form on a rocky substrate **D** macrophotograph of holotype in alcohol.

The Pigeon River arises in the Blue Ridge Mountains of western North Carolina and flows to the northwest until its confluence with the French Broad River in eastern Tennessee (Fig. 1A, B). The Pigeon and French Broad rivers are eastern head water tributaries of the Tennessee River. The Pigeon River traverses the Pisgah National Forest, the Cherokee National Forest, and drains much of the northeastern Great Smoky Mountains National Park. From Canton, North Carolina to its confluence with the French Broad River in Tennessee, anthropogenic impacts to the river have been substantial. The Pigeon River was once considered one of the most polluted rivers in North America (Etnier and Starnes 1993). In 1908 a paper mill began production in Canton, upstream of the Tennessee study area. Effluent from this mill contained a mixture of chemicals including dioxin, chloroform, organic halides, phenols, and catechols (Bartlett 1995). The toxicity of this mixture caused a significant loss of biodiversity. All gastropods (10 species), mussels (40 species), and many fish species disappeared from the river (Coombs 2010). Releases of high levels of tannins and lignin resulted in the river water becoming coffee brown in color and emitting a bad odor. Hot water releases from the mill raised water temperature.

For 52 years there was no treatment of the mill’s effluent. The first effort at treatment occurred in 1960 with the removal of settleable solids (University of Tennessee 2021). In 1989 the mill began an effective dioxin control program. Dioxin has not been detected in tested water since 1989 (Coombs et al. 2010). In 1996 aquatic snails were re-introduced. The survival and recolonization of these snails led to the formation of the Pigeon River Recovery Project (PRRP) in 2001 (Coombs et al. 2010). The PRRP which is composed of volunteers from federal and state agencies, industry, and private organizations has the goal of increasing the aquatic biodiversity of the Pigeon River (Coombs 2010). Due to the reintroduction of aquatic organisms by the PRRP the Pigeon River has regained some of its biodiversity

## ﻿Materials and methods

Two sites on the Pigeon River were surveyed by viewing appropriate hard substrates (i.e., rocks and logs) while wading. A total of 71 sponge specimens were collected, four of which were morphologically distinct from all other extant Spongillida of the Nearctic Region. A minimum of 1 human-hour of search time was spent at each collection site. Latitude and longitude were obtained using a Garmin GPSmap 76CSx receiver. Sponges were viewed using a 10× head-band magnifier for the presence of gemmules. If gemmules were found a section of the sponge was collected. Sponges were preserved in 70% ethanol until processed for light microscopy (LM) and scanning electron microscopy (SEM).

To characterize morphotraits and obtain clean spicule preparations for SEM observation and LM slides, excised sponge was dissolved in test tubes containing 65% nitric acid. Once dissolved, the remaining spicules were centrifuged to create a pellet. Pellets were rinsed and centrifuged three times in distilled water, followed by a final rinse and spin in 70% ethanol. Spicules were pipetted on to glass slides for LM analysis and onto stubs for SEM analysis. A glass substratum was placed under the spicules providing a black background in the SEM photomicrographs (Manconi and Pronzato 2000). Skeleton sections, disassociated spicules, entire gemmules, and gemmule cross-sections were sputter coated with gold-palladium using an Anatech Hummer IV (6.2) sputter coater and observed by Leo 982 and Hitachi TM 3000 SEM. Sponge identification was made using the keys of Manconi and Pronzato (2002, 2016) and descriptions of Reiswig et al. (2010), Penney and Racek (1968), and Racek (1969). To determine range, mean ± standard deviation for spicular lengths, widths, and rotule diameters 75 megascleres and gemmuloscleres were measured using the measurement program of the Hitachi TM 3000. Diameters of 10 gemmules were measured. A paired *t*-test analysis, using JMP software, was used to test the null hypothesis of no significant difference between diameters of gemmulosclere rotules.

### ﻿Institutional acronyms

**FW-POR** R. Manconi and R. Pronzato collection, Italy.

**USNM**National Museum of Natural History, Smithsonian Institution, Washington DC, USA.

**WAM**Western Australian Museum, Perth, Western Australia.

## ﻿Systematic account

### ﻿Phylum Porifera Grant, 1836

**Class Demospongiae Sollas, 1885**

**Subclass Heteroscleromorpha Cárdenas, Perez & Boury-Esnault, 2012**

**Order Spongillida Manconi & Pronzato, 2002**

#### Family Spongillidae Gray, 1867

##### ﻿Genus Heterorotula

Taxon classificationAnimaliaSpongillidaSpongillidae

Penney & Racek, 1968

E65388B8-23C1-53F5-8197-6A13B9D162F0

###### Type species.

*Heterorotulacapewelli* (Bowerbank, 1863).

##### ﻿ Heterorotula lucasi

Taxon classificationAnimaliaSpongillidaSpongillidae

Manconi & Copeland sp. nov.

819CAACF-907A-5E7B-AED4-5D3CEBBB6A00

https://zoobank.org/5886FA19-94E5-43D8-8EF6-AB41E0D819F3

Figs 1 , 2 , 3 , 4 , 5 , 6 , 7 , 8 , 9 ; Tables 1 , 2

###### Type locality.

Pigeon River 35.9396; −83.1786, Cocke County, Tennessee, USA.

###### Type data.

***Holotype*.**USNM 1662182, entire specimen, 70% ethanol, coll. J. Copeland, 30 July 2013. ***Paratype*.**USNM 1662183, fragments, 70% ethanol. Pigeon River, Cocke County, Tennessee, USA, 35.9396; −83.1786, coll. J. Copeland, 30 July 2013.

###### Other material.

BMNH 1890.1.9.339, holotype, *Heterorotulacapewelli* type species of genus *Heterorotula*. WAM Z27997, FW-POR 881, *Heterorotulamultiformis* (Weltner, 1910) (Australia), WAM Z98316, FWPOR 883, Heterorotulacf.multidentata (Weltner, 1895) (Australia).

###### Diagnosis.

*Heterorotulalucasi* sp. nov. is characterized by (a) gemmuloscleres as spiny birotules with flat rotules (distal and proximal) of significantly different diameters with crenulated/notched to shallowly incised margins, (b) absence of skeletal microscleres, (c) skeletal acanthoxeas spiny, and (d) free (not sessile) gemmules.

###### Etymology.

The specific epithet *lucasi* refers to *lux* meaning light in Latin and is dedicated to Lucas Edward Copeland whose enthusiasm and love for the natural history of the forests and streams of the Appalachian Mountains of Tennessee resulted in many wonderful discoveries.

###### Description.

**Figure 2.**
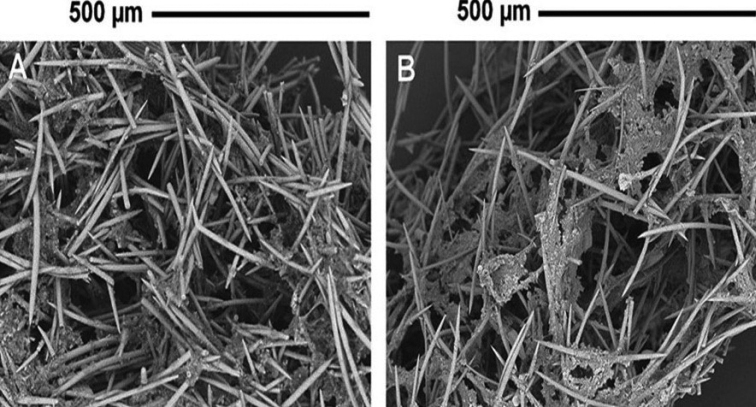
*Heterorotulalucasi* sp. nov. from the Pigeon River, Tennessee (type locality). Micrographs of skeletal network with scanty spongin by SEM**A** multispicular fibres of entosomal area **B** paucispicular fibers of ectosomal area. Scale bar: 500 μm.

Adult sponges with gemmules. No brooded larvae were found. ***Growth form*** (Fig. 1C) encrusting 1–4 mm in thickness and to 16 cm in diameter. ***Surface*** with irregular ridges, hispid from emerging spicules, and with a network of subdermal canals covered by a hyaline dermal membrane. ***Color*** tawny *in vivo* (Fig. 1C), light yellow to light brown in alcohol (Fig. 1D). ***Consistency*** of live sponge soft and fragile. Spongin scanty in skeletal network, arranged as irregular polygonal meshes, to abundant in gemmular theca and basal spongin plate. ***Ectosomalskeleton*** of slender megascleres in paucispicular fibres, with no special architecture (Fig. 2B) supports the dermal membrane. ***Choanosomalskeleton*** (Fig. 2A) as a network of multi-spicular fibres, with scanty spongin. ***Basal spongin plate*** well developed.

**Figure 3.**
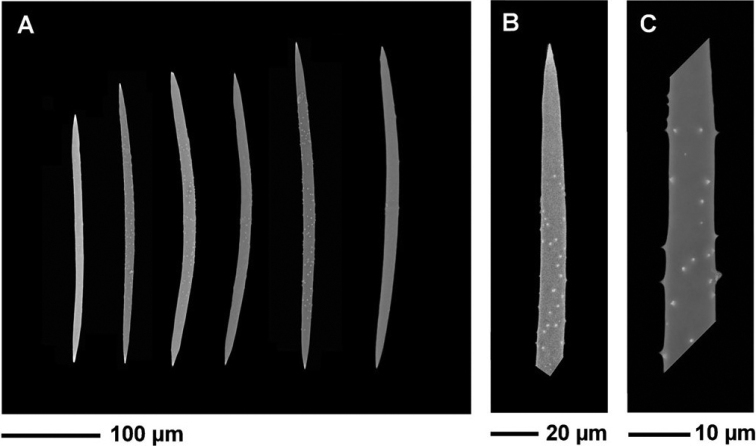
*Heterorotulalucasi* sp. nov. from the Pigeon River, Tennessee (type locality). Micrographs of skeletal megascleres by SEM**A** acanthoxeas fusiform from nearly spineless to variably spined excepts towards the tips **B** detail of an oxea smooth only at the tip **C** spiny shaft of an acanthoxea; scattered spines and microspines on shaft. Scale bars: 100 μm (**A**); 20 μm (**B**); 10 μm (**C**).

***Megascleres*** (Figs 2, 3) fusiform acanthoxeas 223.1–335.0 μm (276.8 ± 24.7) in length and 7.7–13.7 μm (10.9 ± 1.0) in width, slightly curved, with variably dense spines except towards the variably pointed tips, to less frequently nearly spineless. Acanthoxeas shaft from slender in ectosomal area to stouter in the endosome. ***Microscleres*** absent.

**Figure 4.**
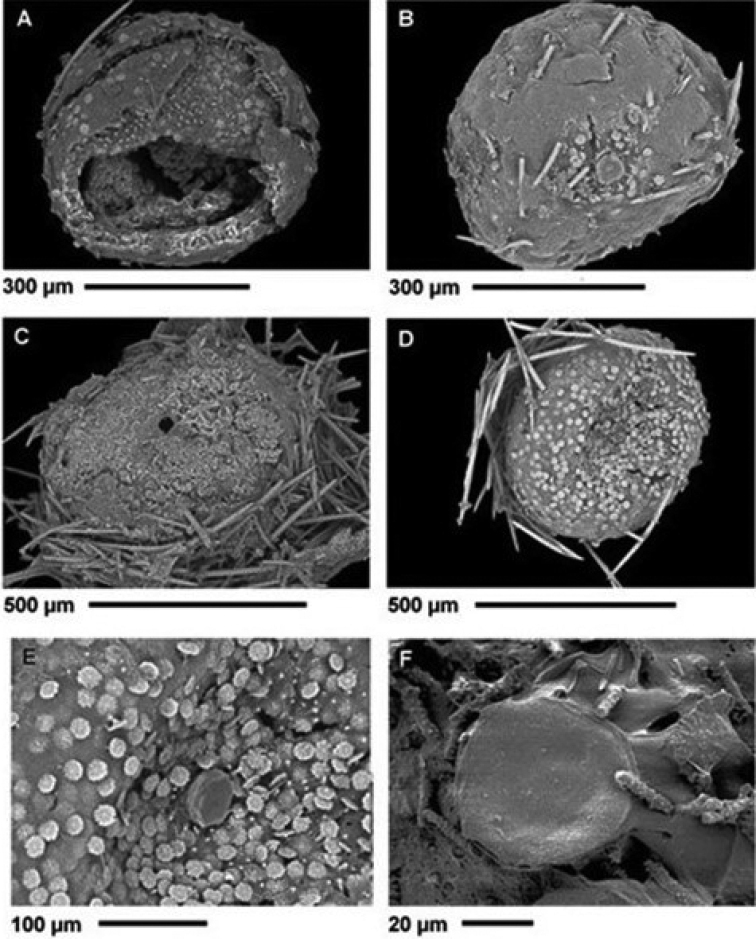
*Heterorotulalucasi* sp. nov. from the Pigeon River, Tennessee (type locality). Micrographs of gemmules by SEM**A** gemmule with multilayered theca armed by radial gemmuloscleres around the central cavity bearing a mass of totipotent cells **B** outer layer at theca surface with a few emerging rotules of gemmuloscleres, acanthoxeas of the cage and sligtly elevated foramen closed by a membrane **C**theca of an aged gemmule covered by diatoms frustules, surrounded by the spicular cage of acanthoxeas and outer layer with open foramen after hatching **D**theca with a fragment of spicular cage and outer layer armed by dense distal rotules of gemmuloscleres **E** fibrous structure of outer layer with sligtly elevated foramen closed by a membrane before hatching **F** fibrous structure of outer layer around foramen and sparse broken shafts of gemmuloscleres. Scale bars: 500 μm (**C, D**); 300 μm (**A, B**); 100 μm (**E**); 20 μm (**F**).

***Gemmules*** (Fig. 4) scattered in skeletal network, subspherical, 448–613 μm (528 ± 55.9) in diameter. Foramen simple (Fig. 4E, F) with smooth undulate margins, slightly elevated above gemmule surface. ***Gemmularcage*** (Fig. 4C, D) of acanthoxeas with small spines.

**Figure 5.**
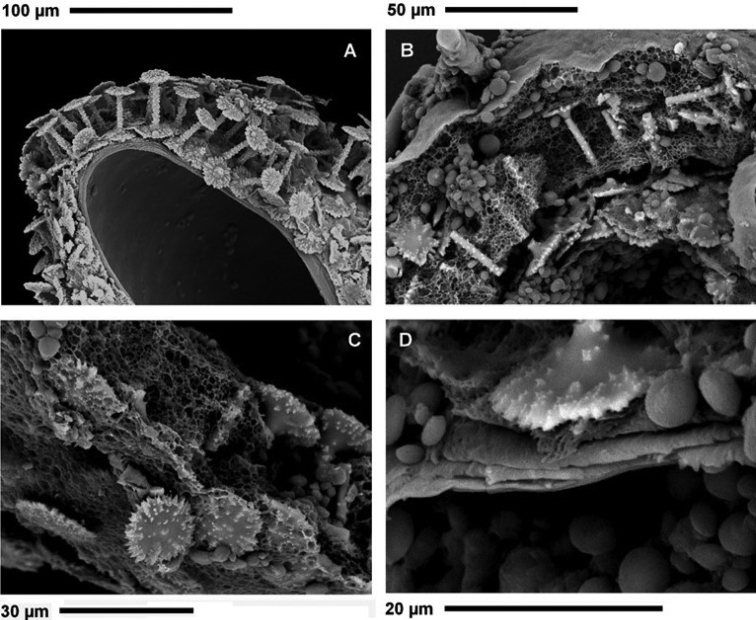
*Heterorotulalucasi* sp. nov. from the Pigeon River, Tennessee (type locality). Micrographs of gemmular theca cross sections by SEM**A**theca with the radial monolayer of short and long spiny birotules bearing crenulated/notched margins of rotules. proximal, partly overlapping, rotules adherent to the multilayered inner layer (cross section, outer layer, and pneumatic layer not evident) **B** trilayered theca with short and long spiny birotules embedded in the fibrous/chambered pneumatic layer in between the smooth outer layer and the multilayered inner layer, **C** radial birotules in the fibrous to chambered pneumatic layer **D** proximal rotule with crenulated/notched margins adherent to the multilayered inner layer surrounding the central gemmular cavity containing totipotent cells. Scale bars: 100 μm (**A**); 50 μm (**B**); 30 μm (**C**); 20 μm (**D**).

***Gemmulartheca*** trilayered ~50 μm in thickness (Fig. 5). **Outer layer** fibrous to compact with distal rotules more or less embedded (Fig. 5A, B, D). **Pneumatic layer** well developed and thick, ranging in the same gemmule from mainly fibrous to chambered network of irregularly polygonal meshes of variable size (Fig. 5B, C). ***Inner layer*** (Fig. 5A, D) multilayered of 3–5 layers of compact spongin. ***Gemmuloscleres*** radially embedded (Fig. 5A, B) as a dense monolayer in pneumatic layers of theca, with distal smaller rotules partly covered by outer layer and proximal larger rotules, partly overlapping one to each other, not embedded into inner layer (Fig. 5A, B).

**Figure 6.**
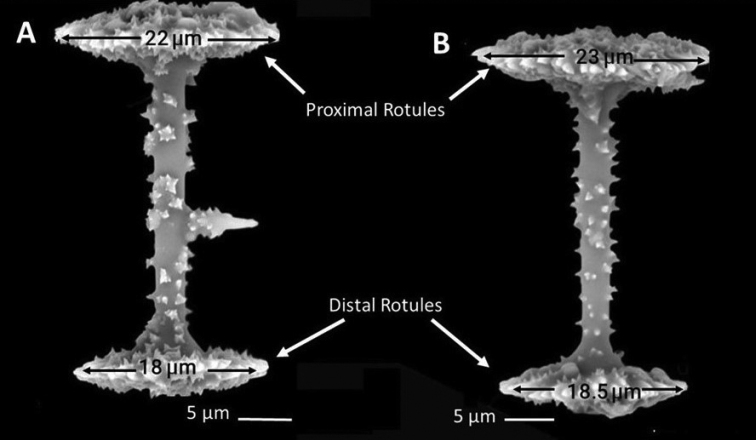
*Heterorotulalucasi* sp. nov. from the Pigeon River, Tennessee (type locality). Size differences in proximal and distal rotules. Scale bars: 5 μm (**A**); 5 μm (**B**); scale bars are slightly different in length.

**Figure 7.**
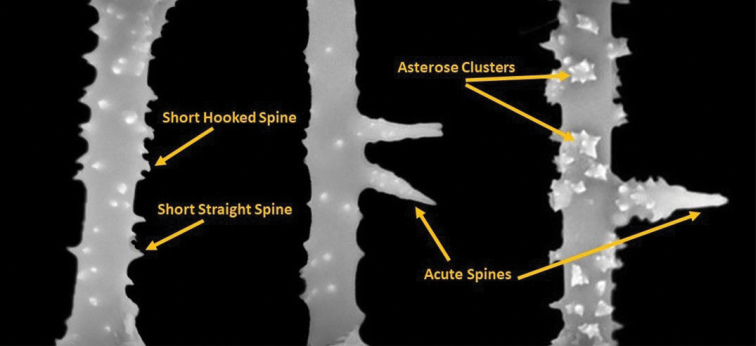
*Heterorotulalucasi* sp. nov. from the Pigeon River, Tennessee (type locality). Spine morphology of shafts of gemmuloscleres by SEM.

**Figure 8.**
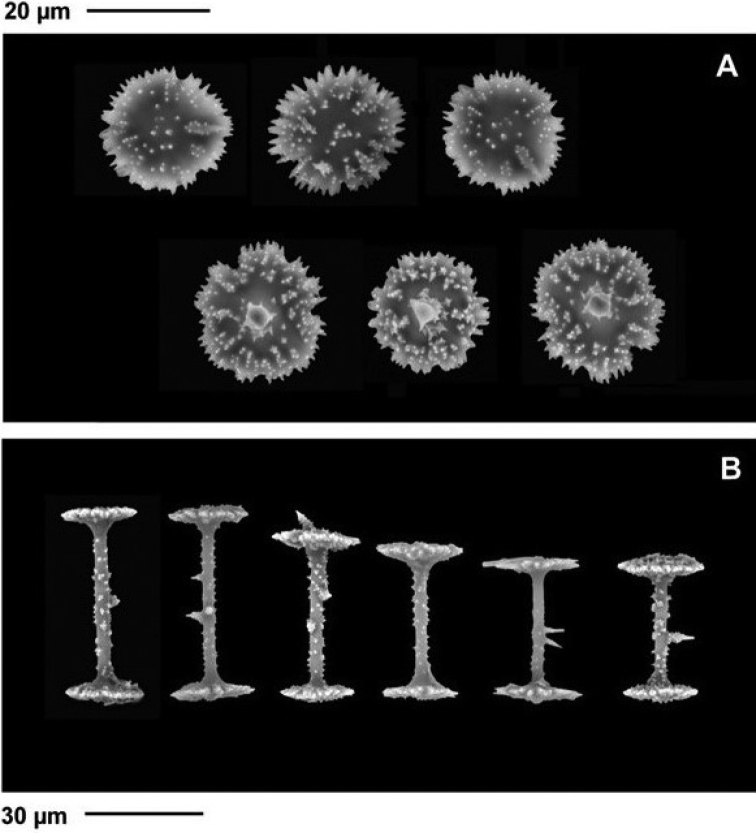
*Heterorotulalucasi* sp. nov. from the Pigeon River, Tennessee (type locality) **A** crenulated margins and miceospines of rotules and gemmulosclers **B** variation in size and spines of gemmuloscleres. Scale bars: 30 μm (**B**); 20 μm (**A**).

***Gemmuloscleres*** (Figs 6–8) slender birotules 19.8–48.6 μm (35.1 ± 5.5) in length, with narrow spiny shaft 2.7–4.4 μm (3.3 ± 0.3) in width. Shaft spines of three types (a) simple, short curved to straight, smooth spines, (b) spines with tips arranged in asterose clusters (microspines in rosettes), and (c) large, acute spines up to 3 μm long bearing secondary microspines (Fig. 7). ***Rotules*** flat with crenulated/notched to shallowly incised margins and both rotules inner and outer surfaces bearing numerous microspines sometimes in radial tows (Fig. 8A) of significantly different diameters *t* (74) = 18.67, *p* < 0.00005) (Fig. 6). Large proximal rotules 19.4–24.4 μm (21.6 ± 1.1) in diameter, small distal rotules 16.6–21.7 μm (18.9 ± 1.1) in diameter.

###### Habitat

**(Fig. 1B).** All four specimens of *Heterorotulalucasi* sp. nov. were found bearing gemmules on the underside of shaded rocky substrate in shallow running water at an altitude of 325 m. Associated aquatic community was composed of diatoms, Spongillidae of three genera/species *Heteromeyenialatitenta* (Potts, 1881), *Radiospongillacerebellata* (Bowerbank, 1863), and *Trochospongillahorrida* (Weltner, 1893); Mollusca of two families Pleuroceridae and Corbiculidae, Diptera larvae of the families Chironomidae and Simuliidae; Ephemeroptera larvae of two families Baetidae and Heptageniidae; Trichoptera larvae of family Hydropsychidae, and Odonata larvae of family Gomphidae. The climate of the study area is temperate having an average annual rainfall of 112 cm. In addition to rain events, flow rates of the Pigeon River are influenced by water releases from a hydroelectric dam in Haywood County, North Carolina. Over its 113 km course the Pigeon River drops from an elevation of 800 m at Canton, North Carolina to 310 m at its confluence with the French Broad River near Newport, Tennessee.

###### Geographic range.

*Heterorotulalucasi* sp. nov. is only known from Pigeon River (type locality, 35.9396, −83.1786) in Tennessee. The location of this single population lays far outside the previously known range of the genus.

## ﻿Discussion

**Figure 9.**
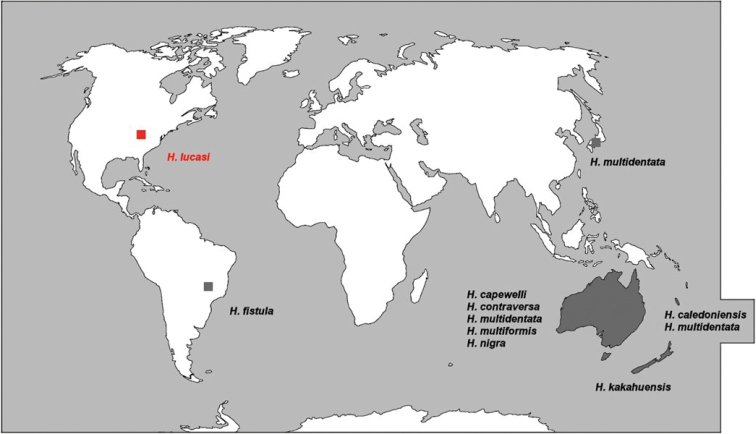
Disjunct biogeographic pattern of genus *Heterorotula* (Porifera, Spongillida, Spongillidae) with enclaves in Nearctic and Neotropical regions. The Nearctic *H.lucasi* sp. nov. from Tennessee is indicated in red. *Heterorotulamultidentata* is considered an alien species in Japan.

The genus *Heterorotula* was erected by Penney and Racek (1968: 96–97) to allocate five species from the Australasian Region (Fig. 9), i.e., *Heterorotulacapewelli* (Bowerbank, 1863) as type species, *Heterorotulakakahuensis* (Traxler, 1896), *Heterorotulamultidentata* (Weltner, 1895), *Heterorotulamultiformis* (Weltner, 1910), and *Heterorotulanigra* (Lendenfeld, 1887) for genus transfer (see Bowerbank 1863; von Lendenfeld 1887; Weltner 1895, 1910; Traxler 1896; Penney and Racek 1968; Rützler 1968) from genera *Ephydatia* Lamouroux, 1816, *Spongilla* Lamarck, 1816, *Tubella* Carter, 1881, and *Meyenia* Potts, 1882 with junior synonyms, e.g., *Spongillacapewelli* Bowerbank, 1863; *Spongillasphaerica* Lendenfeld, 1887, *Tubellanigra* Lendenfeld, 1887, *Ephydatiamultidentata* (Weltner, 1895), *Tubellamultidentata* Weltner, 1895, *Ephydatiakakahuensis* Traxler, 1896, *Ephydatialendenfeldi* Traxler, 1896, *Ephydatiamultiformis* Weltner, 1910, and *Ephydatianigra* Gee, 1931.

A year later, *Heterorotulacaledonensis* (Rützler, 1968) was described as Ephydatiamultidentatef.caledonensis by Rützler (1968). In his synopsis on the Australian freshwater sponges, Racek erected *Heterorotulacontraversa* (Racek, 1969). Later, Volkmer-Ribeiro and Motta (1995) described *Heterorotulafistula* from Brazil. Additionally, *H.multidentata* was reported in Japan (Palaearctic) by Masuda (1998, 2006) and Ishijima et al. (2008) as an alien species (Fig. 9).

The reason given by Penney and Racek (1968: 96–97) to justify the erection of the new genus was that “The peculiar structure and often greatly varying length of the gemmoscleres, so typical for this genus.” They also stated that: “*Heterorotula* is more closely related to *Ephydatia* than to any other genus.” Effectively not all the *Heterorotula* species share very close gemmuloscleres morphologies and, in some cases, a correct identification is not easy.

As for the morphotraits of *Heterorotula* species, Penney and Racek (1968), Racek (1969), and Manconi and Pronzato (2002) focused on length differentials of gemmuloscleres, morphologies of rotule margins, and diameter differences between proximal and distal rotules. An exhaustive and clear key together with images was provided by Racek (1969: 292–293) for morphological divergencies among the five Australasian *Heterorotula* species. As for the main diagnostic traits of the two genera *Heterorotula* and *Ephydatia*, see the following key.

### ﻿Key to the genera *Ephydatia* and *Heterorotula*

**Table:** 

1	Gemmuloscleres only birotules of one length (short shaft) radially arranged; rotule margins evidently indented and spiny with marginal teeth deep and straight; pneumatic layer chambered; microscleres absent; megascleres as microspiny and/or smooth oxeas	***Ephydatia***
–	Gemmuloscleres only birotules of varying length with rotules (short to long shaft) radially arranged; rotules frequently of different diameter, margins crenulated/incised, with many small spines; pneumatic layer fibrous to chambered; microscleres absent; megascleres as microspiny and/or smooth oxeas	***Heterorotula***

As for the only record of the genus known until now from Americas, *Heterorotulafistula* is an intriguing species for its biogeographic location and palaeoecology. It is recorded as a fossil/subfossil taxon from spongolites remains in sedimentary deposits of permanent and temporary freshwater bodies of the subequatorial Brazilian areas of Rio Grande do Norte, Goiás Mato Grosso do Sul and Minas Gerais in the Neotropical Region (Volkmer-Ribeiro and Motta 1995; Machado et. al. 2014, 2016; Docio et. al. 2021). Although its subfossil condition cannot support its status as either a true living species or a recently extinct one, the Neotropical enclave of this species enlarged the disjunct biogeographic pattern of *Heterorotula* to the western hemisphere (Fig. 9).

In any case, the very different geological histories of the Gondwanian, Australasian, and Neotropical regions, after the Pangea breakup (~175 Mya), do not provide an explanation for this biogeographic pattern of *Heterorotula*. This task is made more difficult because very few ancient fossils of freshwater sponges have been found (Pronzato et al. 2017; Samant et al. 2021).

Results of a comparative analysis of *H.lucasi* sp. nov. with congeneric species, on the basis of museum collection and original descriptions, revealed that it significantly diverges from all other species of *Heterorotula* by a few robust characters.

The growth form, oscules, color, and skeletal architecture are not diagnostic morphotraits for the new species and for genus *Heterorotula*, as for most Spongillida. The absence of skeletal microscleres is shared by all species of the genus.

The trait “megascleres as fusiform oxeas prevalently spiny to nearly spineless” is partly and differently shared with some *Heterorotula* species. The comparsion between the type species *H.capewelli* and *H.lucasi* sp. nov. revealed that the megascleres of the former (195–300 × 13–18 μm) are stout oxeas predominantly smooth with few evident scattered spines, while those of the new species are slimmer oxeas predominantly and abundantly microspiny. The oxeas lengths range reported by Penney and Racek (1968) for other species is from *H.nigra* (224–360 × 7–13 μm), *H.multidentatae* (284–320 × 10–18 μm), *H.multiformis* (330–420 × 13–20 μm) to *H.kakahuensis* (185–260 × 9–12 μm). Length and width measurements for megascleres, gemmuloscleres, and gemmules of the known living species of *Heterorotula* are presented in Table 2.

**Table 2.** Length and width ranges, in microns, for spicules and gemmules of living species of *Heterorotula*.

Species	ML	MW	LBL	SBL	BSW	PRD	DRD	GW
*H.capewelli*	195–330	13–18	38–52	34–45	3–4	24–45	20–23	510–560
*H.nigra*	224–360	7–13	56–73	35–48	2–4	13–16	10–14	230–360
*H.multidentata*	284–320	10–18	64–84	32–48	4–6	19–22	17–20	490–580
*H.kakahunesis*	185–260	9–12	30–42			17–22		380–540
*H.multiformis*	330–420	13–20	35–52	24–44	2–4	14–24	14–18	480–680
*H.caldonensis*	250–410	8–20	35–85		4	19–26	18–22	450–600
*H.controversa*	210–395	12–21	21–57		3–4	20–24	18–21	470–590
***H.lucasi***	223–335	7.7–13.7	19.8–48.6		2.7–4.4	19.4–24.4	16.6–21.7	448–613

Range values positioned between columns indicate no separation into two distinct size classes for that variable. Legend: ML megasclere length; MW megasclere width; LBL large birotule length; SBL small birotule length; BSW birotule shaft width; PRD proximal rotule diameter; DRD distal rotule diameter, GW gemmule width.

The gemmule architecture and dimensions and its morphotraits, i.e., spicular cage, pneumatic layer, and foramen matches the genus range. The gemmuloscleres of *H.lucasi* sp. nov. are unique. Both rotules are finely spined and with crenulated/incised margins. The slim shaft of gemmuloscleres displays spines moderately long and variable in length.

Only the type species of the genus, *H.capewelli*, shares with *H.lucasi* sp. nov. the trait “crenulated rotules.” The term “crenulated” refers to the New Latin term *crenulatus*, from *crenula*, diminutive of Medieval Latin *crena* (notch) was introduced by Racek (1969: 293) and meaning “Having a margin contour with shallow, usually rounded notches and projections; finely notched or scalloped.” However, the two species diverge for gemmulosclere shaft morphologies, *H.capewelli* has smooth to finely granulated shafts whereas the shafts of the new species are variously spined. All other species of the genus show gemmuloscleres with margins of rotules variously indented/incised and with variably long to very long shafts.

Among freshwater sponges gemmuloscleres birotules are shared by several genera having a wide range of rotule morphotraits, e.g., flat, curved, umbonate, indented, hooked, smooth, and variably spiny/tubercled. In *Heterorotula*, the morph “rotules with margins only crenulated” seems to be shared by only two species, *H.capewelli* and *H.lucasi* sp. nov. However, also the margins of the rotules of the Neotropical *Ephydatiafacunda* (Pinheiro et al. 2004) resemble those of the new species but diverges from it for the shaft morphology, whereas the Middle Eocene fossil E.cf.facunda from Canada totally diverges from *H.lucasi* sp. nov. in the outline of the birotules (Pisera et al. 2016).

In contrast, it is noteworthy that the southeastern Nearctic harbors *Ephydatiamillsi* (Potts, 1887), which is exclusively endemic to the type locality at Sherwood Lake near DeLand, Florida (Potts 1887; Penney and Racek 1968; Manconi and Pronzato 2016). This aberrant species, which does not match the diagnosis of *Ephydatia*, was considered “eventually to be an ecomorph of one of the more widely distributed *Ephydatia* species” (Penney and Racek 1968: 96) because it displays the morph “spiny, flat rotules with lacinulate margins” (Penney and Racek 1968; Reiswig et al. 2010). *Ephydatiamillsii* must still be considered as rather insufficiently known.

The gemmuloscleres of *H.capewelli* are birotules with a slender, smooth shaft bearing sometimes few spines. Rotules with unequal diameter are flat with small spines and margins irregularly crenulated with rare teeth. In comparison the birotules of *H.lucasi* sp. nov. are smaller and with margins of rotules irregularly crenulate/incised and evidently spiny, with also a stouter shaft bearing abundant rosetta-shaped microspines and rare long spines. Diameter of rotules is unequal.

Summarizing, the new species is characterized by slimmer megascleres that are predominantly and abundantly microspiny to nearly spineless; smaller gemmuloscleres that are abundantly microspiny on rotules and the stout shaft (the latter with also spines in rosettas), and a very distant disjunct geographical range.

This biogeographic enclave in Tennessee contributes to the understanding of the origin of Nearctic inland water biodiversity, biogeographical patterns, and morphological adaptive traits in Nearctic sponges. The present record matches the recent discovery in Tennessee of *Cherokeesiaarmata* Copeland, Pronzato & Manconi, 2015, the first living Nearctic Potamolepidae (Copeland et al. 2015). Data confirm that the Appalachian region (Ordovician–Permian origin) of Tennessee and, in general, of southeastern North America have high levels of diversity and endemicity (Curtin et al. 2002; Hodkinson 2010; Erlandson et al. 2021).

With the present record of a new species in Tennessee, the biogeographic pattern of the genus *Heterorotula* matches the Australasian–Pacific Islands–Neotropical regions in the southern hemisphere (Australia *n* = 5 species; New Caledonia *n* = 2; New Zealand *n* = 1; Brazil *n* = 1 species), with one spot in a Nearctic enclave (*n* = 1 species, *H.lucasi* sp. nov.) and an alien species in the insular easternmost Palaearctic (Japan *n* = 1) (Fig. 9) (Manconi and Pronzato 2008; Manconi et al. 2016; Pronzato and Manconi 2019; de Voogd et al. 2022).

Not considering the Japanese record of an alien species, the scenario that arose with the unexpected discovery of a *Heterorotula* species in the southwestern Laurasian Nearctic (North America), and the subsequent tripartite geographic range, is anomalous but not unique to and shows a similitude with, for example, marsupials of which the majority of species is strictly Australasian but having some key species recorded from the Neotropical and Nearctic regions (e.g., Opossum, *Didelphisvirginiana* (Voss 2022)).

## Supplementary Material

XML Treatment for Heterorotula

XML Treatment for Heterorotula lucasi
